# The mermithid species *Isomermis lairdi* (Nematoda, Mermithidae), previously only known in Africa, found in Europe

**DOI:** 10.3897/zookeys.454.7577

**Published:** 2014-11-12

**Authors:** Denis Gradinarov

**Affiliations:** 1 Department of Zoology and Anthropology, Faculty of Biology, Sofia University “St. Kliment Ohridski”, 8 Dragan Tsankov Blvd., 1164 Sofia, Bulgaria

**Keywords:** Entomoparasitic nematodes, morphology, taxonomy, parasite ecology, distribution, *Simulium ornatum*, disease vectors, black fly control, Bulgaria

## Abstract

The present work contributs to the knowledge on the aquatic mermithids (Nematoda, Mermithidae) occurring in black flies – an insufficiently studied group of parasitic nematodes. *Isomermis lairdi* Mondet, Poinar & Bernadou, 1977, described from larvae of *Simulium damnosum* Theobald, 1903 in Western Africa, is reported to occur in Bulgaria. The species was isolated from larvae of *Simulium ornatum* Meigen, 1818 in a local population of simuliids in a mountain stream near Jeleznitsa Village, Sofia district. Postparasitic juveniles of mermithids were obtained from the hosts and reared to the adult stage. The species was identified by morphological and morphometrical characters of postparasitic juveniles, and of male and female individuals. In the summer of 2012 a relatively high rate of mermithid infection in a local host population was detected (prevalence up to 44.1%). In August of the next year host abundance had considerably declined and other simuliid species, *Simulium variegatum* Meigen, 1818 and *Simulium reptans* (Linnaeus, 1758), predominated in the investigated locality. In West Africa, *Isomermis lairdi* is considered to be a potential biological agent for reducing the population density of the *Simulium damnosum* complex – the main vector of human onchocerciasis. In Europe, species of the *Simulium ornatum* complex are among the vectors of onchocerciasis of cattle and deer. The mermithids presumably play a certain role in the epidemiology of these diseases. A brief discussion on the taxonomy of the genus *Isomermis* Coman, 1953, and of the feasibility of molecular methods in mermithid taxonomy is provided. The species *Isomermis lairdi* is reported for the first time from Europe.

## Introduction

Mermithids (Nematoda, Mermithidae) are lethal parasites of arthropods, mainly insects. Species of at least 15 different orders of insects are among the hosts of the family (Nickle 1972). Mermithids occurring in black flies (Diptera, Simuliidae) are an insufficiently studied group with problematic taxonomy (Molloy 1981, St-Onge et al. 2008). Descriptions of many species are not satisfactory and many described species are considered as *species inquirendae* (Curran and Hominick 1981, Poinar and Takaoka 1979). The adult nematodes, essential for a correct morphological identification (Poinar 1979), need in most cases to be obtained by laboratory rearing of emerged postparasitic juveniles. Thus, morphology-based taxonomy requires significant time and effort. Recently, molecular methods have been introduced in the taxonomy of the group (St-Onge et al. 2008, Crainey et al. 2009).

Mermithids may play an important role in the regulation of population densities of simuliid hosts (Rubzov 1974, Molloy 1981, Crainey et al. 2009), but problems in taxonomy and insufficient data on the biology and ecological requirements (St-Onge and Charpentier 2008) complicate the use of mermithids for black fly control. The difficulties in the identification of immature stages of the hosts, as well as unsolved taxonomic problems in the Simuliidae (Molloy 1981, Adler and Crosskey 2013) further discourage research on the host–parasite relationships within this group.

The aim of the present study was to *i*) perform identification of newly isolated mermithids from a local population of Simuliidae in Bulgaria, *ii*) analyze the taxonomic position of these species within the genus and their geographical distributions, and *iii*) provide original data on the rate of mermithid infection in the investigated host population and discuss the host–parasite relationships at population level.

## Materials and methods

Larvae and pupae of Simuliidae were collected from (1) the Selska Reka River (mountain stream) just above Jeleznitsa Village, Vitosha Mts. (42°32.04'N; 23°21.79'E, 1050 m a.s.l.), and (2) from a channel which diverts the water from the river and supplies barrage ponds in the village square (42°32.05'N; 23°21.91'E, 1030 m a.s.l.). The sampling was carried out in July-September of 2012 in both localities and in August-September of 2013 in the second locality only. Denotations of nematode individuals used for measurements and species identification are: July (25.07., 1♀), August (03.08., 4♂♂, 1♀, 1♂J, 3♀♀Js; 12.08., 2♂♂, 2♀♀, 1♂J) and September (22.09., 1♂J) of 2012.

Simuliids were collected along with grasses and branches of trees which were dipping in the water. The host larvae were kept in shallow dishes with tap water and were examined periodically for the emergence of postparasitic juveniles of mermithids (Rubzov 1974). Emerged nematodes were placed in Petri dishes with tap water and maintained in the cold (5–7 °C) (method after Camino 1994). The adults were fixed in 4% formaldehyde, transferred to glycerol (simple evaporation method, after Poinar 1975), and mounted on microscopic slides with paraffin rings. The measurements were performed at magnifications of 10×20 and 10×40 using a light microscope (Olympus BX41). The pictures were taken with a digital camera (Olympus Color View I). The prepared microscope slides with mermithids have been deposited in the collection of the Department of Zoology and Anthropology at Sofia University (slides M-VTM: 1-16).

Mermithid identification was performed according Rubzov (1972, 1974). Original descriptions of closely related (Welch 1962, Rubzov 1968) or later described species (Mondet et al. 1977, Poinar and Takaoka 1979, 1981, 1986, Camino 1987, 1994) were also used. The conclusions concerning diagnostic characters in mermithid taxonomy, as suggested by Curran and Hominick (1981), were considered as well.

The rate of mermithid infection is given for the second locality. Host larvae of middle and late instars (body length of 3 mm or more) were examined individually under the stereomicroscope for the presence of parasitic juveniles of nematodes. In suspicious cases host larvae were dissected. Black flies were determined by larvae and pupae (after Yankovsky 2002, Jedlicka et al. 2004, etc.). Collected pupae were used to facilitate identification and to detect the presence of the species in the locality. To confirm the identification, examined specimens were compared with the reference collections of Dr Stanoy Kovachev, held at the Department of Zoology and Anthropology, Sofia University. Prepared microscopic slides with host parts, used for identification, as well as the host larvae with parasitic juveniles of nematodes, fixed in 4% formaldehyde or 70% alcohol, are deposited in the same institution. Nomenclature of Simuliidae is after Adler and Crosskey (2013).

## Results

**Figure 1.**
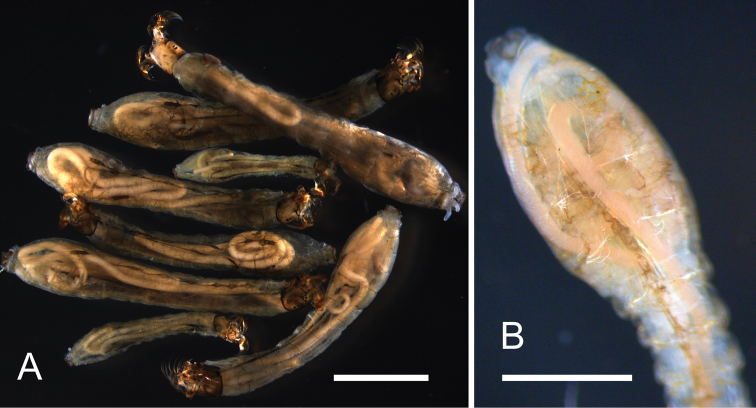
Larvae of *Simulium ornatum* with parasitic juveniles of *Isomermis lairdi*, Selska Reka River. **A** Fixed in 70% alcohol material, 03.08.2012 **B** Live host larva, 12.09.2013. Scale bars: **A:** 2 mm; **B:** 1 mm.

Larvae of Simuliidae infected with mermithids were established in July, August and September of 2012 and August and September of 2013. Mermithids were found in both localities examined mainly in larvae of *Simulium ornatum* Meigen, 1818 (Fig. 1A, B). In the second locality *Simulium ornatum* was clearly predominant during the sampling period of 2012 (Table 1). The estimated rate of its infection with mermithids in late July and August varies from 1.8% to 44.1%. Only nine larvae and one pupa of *Simulium variegatum* Meigen, 1818 were present in quantitative samples, while *Simulium reptans* (Linnaeus, 1758) was detected by a single pupa, collected on 25 July. In summer and early autumn of 2013 four species of Simuliidae were found in the investigated locality. A considerable decline in the population density of *Simulium ornatum* was observed in August, when *Simulium variegatum* and *Simulium reptans* clearly dominated the samples. The infection rate of *Simulium ornatum* in all three samples of August and September, despite the small number of host larvae, was still high (60–81.1%).

**Table 1.** Simuliid species and the rate of their infection with mermithids in the supply channel of the Selska Reka River, Vitosha Mts., July – August of 2012 and August – September of 2013.

Date	Simuliids	Number larvae	Infected larvae	
			N	%
25.07. 2012	*Simulium ornatum*	179	79	44,1
	*Simulium variegatum*	7	1	14,3
	Total (% infected)	186	80	(43,0)
03.08. 2012	*Simulium ornatum*	391	150	38,4
	*Simulium variegatum*	2	0	0,0
	Total (% infected)	393	150	(38,2)
12.08. 2012	*Simulium ornatum*	239	22	9,2
25.08. 2012	*Simulium ornatum*	340	6	1,8
16.08. 2013	*Simulium variegatum*	182	0	0,0
	*Simulium reptans*	50	2	4,0
	*Simulium ornatum*	10	6	60,0
	Total (% infected)	242	8	(3,3)
27.08. 2013	*Simulium variegatum*	110	1	0,9
	*Simulium reptans*	77	3	3,9
	*Simulium ornatum*	11	9	81,1
	Total (% infected)	198	13	(6,6)
12.09. 2013	*Simulium reptans*	50	2	4,0
	*Simulium ornatum*	32	20	62,5
	*Simulium variegatum*	16	0	0,0
	*Simulium* sp.	1	0	0,0
	Total (% infected)	99	22	(22,2)

**Figure 2.**
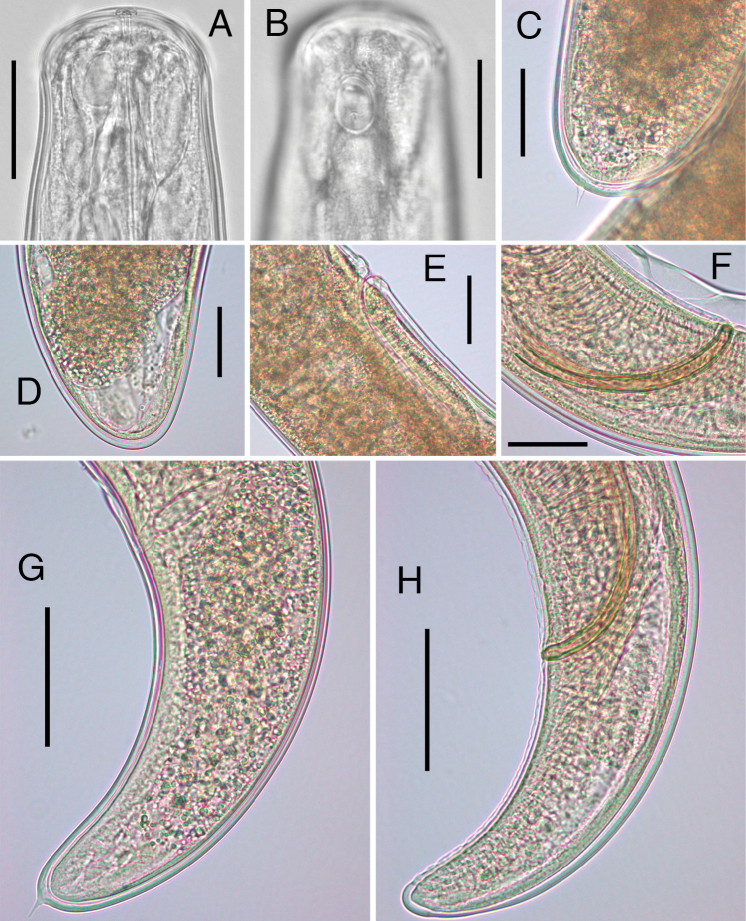
Main morphological characters of *Isomermis lairdi* from Jeleznitsa (**A–H**). **A**, **B** Mature male, anterior region with terminal mouth opening (**A**) and amphid (**B**), lateral view. **C** Female postparasitic juvenile, posterior end with tail appendage **D** Mature female, posterior end **E** Female, vulvar region with vagina **F** Male, spicules **G** Male postparasitic juvenile, tail region with tail appendage **H** Mature male, tail region. Scale bars: **A**, **B:** 50 µm; **C**–**F:** 70 µm; **G**, **H:** 100 µm.

**Figure 3.**
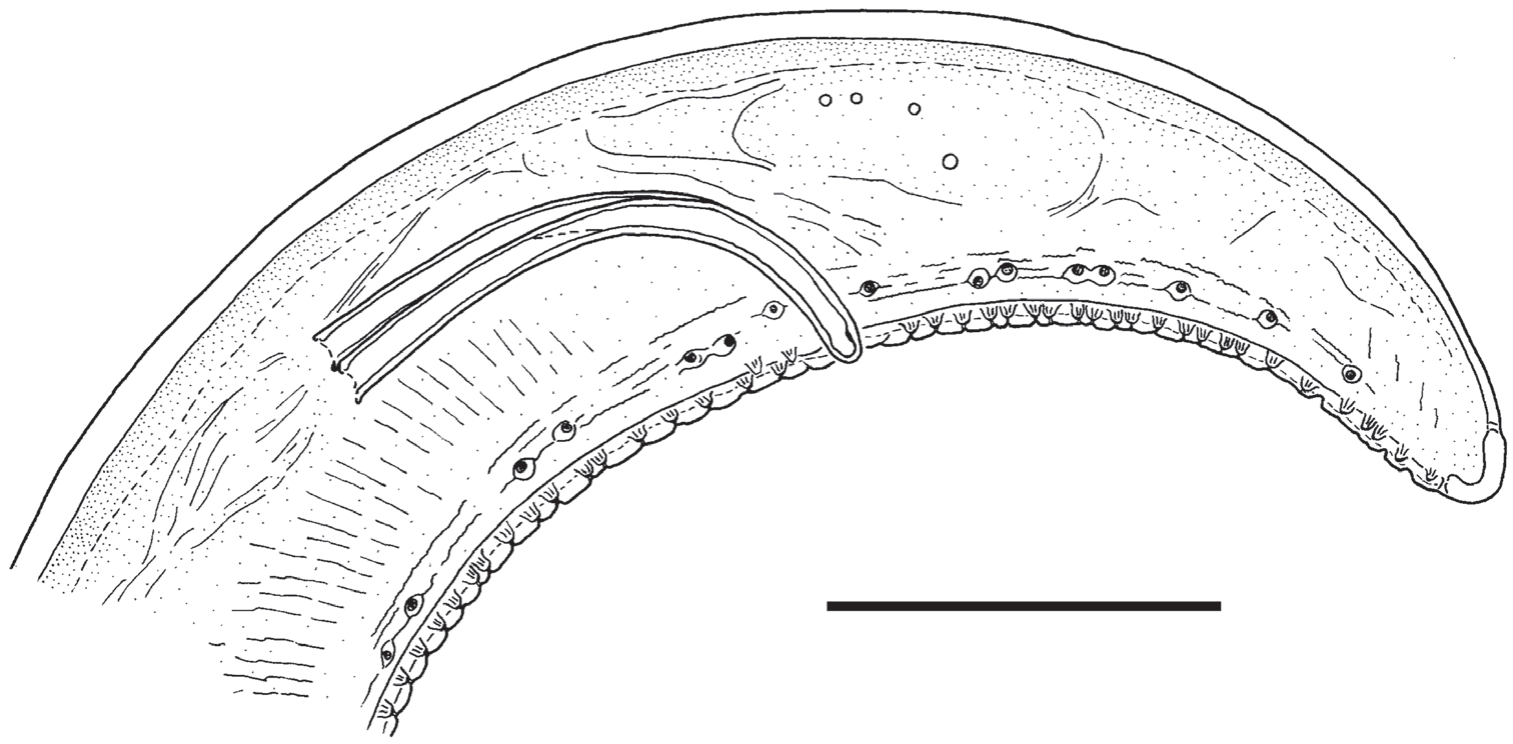
Tail region of male *Isomermis lairdi* from Jeleznitsa with spicules and genital papillae, lateral view. Scale bar: 100 µm.

The emergence of postparasitic juveniles of mermithids was observed only from larvae of *Simulium ornatum* in the summer and early autumn of 2012. Six males and three females, suitable for measurements, were reared to adult stages. The period of maturation and release from the cuticle remains of postparasitic juveniles under rearing conditions was from 20 to 40 days. The attempts to obtain postparasitic juveniles in the second year were unsuccessful. Based on morphological characteristics of postparasitic juveniles, as well as males and females, all mermithids were identified as belonging to genus *Isomermis* Coman, 1953. These characteristics are the presence of: eight hypodermal chords, six cephalic papillae, terminal position of mouth opening (Fig. 2A), oval amphids located posterior to the head papillae (Fig. 2B), short spine-like tail appendages of postparasitic juveniles in both sexes (Fig. 2C, G), S-shaped vagina (Fig. 2E), and paired moderately curved spicules (Figs 2F, H, 3). The main characters of adult specimens are as follows.

*Males* (n=6): body length: 10.81 mm ± 1.51 (9.10–13.26), width of the head at the level of cephalic papillae: 52 µm ± 5 (47–59), width of the body at the level of nerve ring: 93 µm ± 11 (82–105), greatest width of the body: 148 µm ± 16 (127–163), width of the body at cloaca: 123 µm ± 15 (106–141), distance from the head to the nerve ring: 191 µm ± 27 (158–231), tail length: 277 µm ± 37 (238–332), length of amphidial pouch (n=2): 24 µm ± 1 (24–25), width of amphidial pouch (n=2): 17 µm, number of the genital papillae: 18–21 in lateral rows and 56–62 in ventral row, arrangement of the papillae as follows: precloacal – 11–14 in lateral rows and 31–38 in ventral row, postcloacal – 7–8 in lateral rows and 23–26 in ventral row, length of spicules (measured along median): 203 µm ± 10 (191–218), length of spicules (measured along chord): 179 µm ± 8 (168–193). Spicules yellowish-colored, clearly separated at the base and close towards the tip, with elongate distal and arcuate proximal part.

*Females* (n=3): body length: 17.44 mm ± 3.95 (12.96–20.42), width of the head at the level of cephalic papillae: 71 µm ± 5 (67–77), width of the body at the level of nerve ring: 113 µm ± 19 (94–131), greatest width of body: 251 µm ± 39 (208–285), width of the body at vulva: 233 µm ± 42 (186–267), distance from head to the nerve ring: 191 µm ± 26 (171–220), width of the body at posterior end of the trophosome: 111 µm ± 5 (106–116), length of vagina: 219 µm ± 22 (205–245), width of vagina: 54 µm ± 2 (52–57), length of amphidial pouch (n=1): 20 µm, width of amphidial pouch (n=1): 16 µm, V%: 52.1 (50.2–54.8). Vagina is slightly curved, with the first bend extended posteriorly.

Color of the trophosome of the living individuals of both sexes varies from pale pink to brownish red, more intense in parasitic and postparasitic juveniles (Fig. 1B).

After a detailed comparison with the original descriptions of the species the mermithids were identified as *Isomermis lairdi* Mondet, Poinar & Bernadou, 1977, originally described from West Africa. The main reasons for the identification were good conformity with most morphometric characters of the species, the compliance of morphology of the caudal region of the males with the figure in the original description, as well as the morphology of caudal appendage of postparasitic juveniles (Mondet et al. 1977). Taxonomic traits of special importance were the general shape and position of the spicules, the number and arrangement of genital papillae (especially in the lateral rows), including their cohesion in pairs, and the morphology of the tail tip of the males (Figs 2F, H, 3).

No other mermithids, except *Isomermis lairdi*, emerged from larvae of *Simulium ornatum* or were received from them at the selective dissections. Sporadic infection of *Simulium reptans* and *Simulium variegatum* larvae (Table 1), as shown by performed dissections, seems to be caused by other mermithid species, possibly belonging to the genus *Gastromermis* Micoletzky, 1923 (indicated by the presence of a longer tail appendage of parasitic juveniles). The poor condition and the early age of the specimens received at dissection, however, did not allow their exact identification.

## Discussion

The genus *Isomermis*, as well as the whole family Mermithidae (Stock and Hunt 2005), is in need of comprehensive revision. Many characters used in species descriptions exhibit significant intraspecific and ontogenetic variability and are not proven to be useful for species separation (Curran and Hominick 1981). Therefore, in many cases the original diagnoses of the species are inconclusive. Camino (1987) accepts as valid approximately 12 species of the genus, without listing them, and describes three new species (Camino 1987, 1994). Species of *Isomermis* have been reported from Europe (Coman 1961, Rubzov 1968, Rubzov and Doby 1970, Katyukha and Lukhovoz 2008), North America (Welch 1962, St-Onge and Charpentier 2008, St-Onge et al. 2008), Central (Poinar and Takaoka 1979, 1981) and South America (Camino 1987, 1994, Ginarte et al. 2003), Africa (Rubzov 1972, Mondet et al. 1977, Maduabum and Iwuala 1990, Crainey et al. 2009) and Asia (Poinar and Takaoka 1986). The host range of the genus includes different species of Simuliidae (Crosskey and Poinar 2002) and rarely Chironomidae (Rubzov 1968, Camino 1987).

*Isomermis lairdi* resembles most closely three species of the genus. These are *Isomermis rossica* Rubzov, 1968, described from Russia, *Isomermis benevolus* Poinar & Takaoka, 1979 from Guatemala and *Isomermis wisconsinensis* Welch, 1962 from North America. Among the diagnostic characters of *Isomermis lairdi* Mondet et al. (1977) point out the strongly S-shaped vagina. At the same time, in our case bending of the vagina is considerably smaller (Fig. 2E) and corresponds to that of the species *Isomermis wisconsinensis* (Welch 1962) and *Isomermis rossica* (Rubzov 1968). Curran and Hominick (1981) showed, however, that the shape and direction of the vagina are ontogenetically variable. In the description of *Isomermis lairdi* a picture of a mature female with formed eggs was presented, while the individuals we have worked with are young females with retained trophosome, and this does not allow this feature to be used as a diagnostic character in our case.

*Isomermis rossica* is the only repeatedly reported species of *Isomermis* from Europe. It was found in different regions of Russia, Belarus, (Rubzov 1968), France (Rubzov and Doby 1970) and Ukraine (Katyukha and Lukhovoz 2008). *Isomeris rossica*, however, possesses spicules of a different shape – without an elongate distal part, the whole spicule being uniformly and relatively strongly curved (Rubzov 1968, 1972). The number of genital papillae in the lateral rows, mentioned in species description of *Isomermis rossica*, is considerably larger (30–40). *Isomeris rossica* was found in larvae of *Simulium erythrocephalum* (De Geer, 1776), *Simulium morsitans* Edwards, 1915, *Simulium rostratum* (Lundstrom, 1911), *Simulium vernum* Macquart, 1826, *Simulium cryophilum* (Rubzov, 1959), *Simulium lundstromi* (Enderlein, 1921), rarely in other simuliids and chironomids (Rubzov 1968, Crosskey and Poinar 2002). Despite the broad host range of the species, Rubzov (1968, 1974) expressly noted that larvae of *Simulium ornatum* are „immune“ to mermithid invasion in long-term surveys in localities with high occurrence of *Isomermis rossica*. Thus this host specialization can in our case be used as an additional diagnostic feature.

*Isomermis lairdi* was described in Cote d’Ivoire from larvae of *Simulium damnosum* Theobald, 1903 (Mondet et al. 1977) and was subsequently reported to occur in Ghana, Togo, Benin (after Crainey et al. 2009) and Nigeria (Maduabum and Iwuala 1990). The hosts of the species are simuliids belonging to the *Simulium damnosum* complex (after Crainey et al. 2009), as well as *Simulium hargreavesi* Gibbins, 1934 (Maduabum and Iwuala 1990). Species from the *Simulium damnosum* complex do not occur in Europe, as the northern border of the distribution of this complex in West Africa passes through Mali and Niger (Adler and Crosskey 2013). At the same time, the southern border of the distribution of the *Simulium ornatum* complex passes through Morocco and Algeria. Thus, no strong geographic isolation between both host complexes exists, which is a precondition for the dissemination of the mermithids. It appears that in different geographic regions *Isomermis lairdi* could parasitize different hosts. Yet Rubzov (1974) suggests the presence of other African species of the genus *Isomermis*, also described from larvae of *Simulium damnosum* – *Isomermis tansaniensis* Rubzov, 1972 in western Europe, despite the absence of the type host. The latest species, however, was described based on juveniles only (Rubzov 1972) and Poinar and Takaoka (1979) considered it as *species inquirendae*.

Simuliids of the *Simulium damnosum* complex are among the main vectors of human onchocerciasis in Africa (Adler 2004). Thus, *Isomermis lairdi* has been considered as a possible biological agent for the control of these simuliids (Crainey et al. 2009). The same authors published sequencing data of the 18S rDNA region of *Isomermis lairdi* (Crainey et al. 2009). However, authors used parasitic juveniles, recovered from the hosts and identified to genus level as material for isolation of DNA samples. This step of the method per se raises serious suspicions about affiliation of the DNA sequences obtained to the species *Isomermis lairdi*. The figures of taxonomic characters of parasitic juveniles presented by these authors are inconclusive. On the first figure, the position of the mouth opening could not be clearly determined. In some species of *Gastromermis*, i.a. *Gastromermis viridis* Welch, 1962 the ventral displacement of the mouth is less pronounced (Welch 1962). The relatively long caudal appendage of the female parasitic juvenile in the second figure also resembles that of some species of *Gastromermis* (Rubzov 1974). *Isomermis lairdi* possesses a considerably shorter and spine-like caudal appendage (Fig. 2 and Mondet et al. 1977). It is therefore not surprising that in the resulting phylogenetic tree, constructed with the obtained sequences, *Isomermis lairdi* sensu Crainey et al. formed a monophyletic group with *Gastromermis viridis*, rather than with the closely related *Isomermis wisconsinensis* (Crainey et al. 2009). Generally, the obtained sequences are unusable for identification of *Isomermis lairdi* and for other taxonomical purposes within the genus. For an accurate molecular characterization of mermithid species, the identification of the material should be performed on adult specimens. For a taxonomic revision of the genus, interbreeding experiments with closely related species would also be helpful. The latter was recommended by Poinar and Takaoka (1979) in the description of *Isomermis benevolus* with respect to *Isomermis lairdi*, as the authors noted the similarities between the two species despite geographical isolation.

According to previous research on simuliid fauna in Bulgaria, *Simulium ornatum* and *Simulium variegatum* are common in the rivers on Vitosha Mts., while *Simulium reptans* is relatively rare (Kovachev 1990). Mermithid parasitism is a possible cause of the decrease of the population density of *Simulium ornatum* in the investigated locality in August of 2013. During the survey period, cases of infection of simuliid larvae, including *Simulium ornatum*, with several microsporidian species, were also observed (D. Gradinarov, D. Pilarska, unpublished data). The impact of microsporidia on the host population, however, does not appear to be significant, because of the relatively low rates of infection detected with similar degrees in the different simuliid species. On the other hand, the cases of epizootics in host populations, caused by mermithid parasitism, are noted and discussed by Rubzov (1974) and Molloy (1981). The high infection rate of single host species can lead to a change in species composition in a local habitat in the subsequent years and the appearance of “substitute species” which may be “immune” to nematode invasion (Rubzov 1974). In our case, such substitute species seem to be *Simulium variegatum* and *Simulium reptans*. In the Palearctic Region, including Europe, black flies of the *Simulium ornatum* complex are known as vectors of *Onchocerca lienalis* (Stiles, 1892) and *Onchocerca skrjabini* Ruchljadev, 1964 (syn. *Onchocerca tarsicola* Bain & Schulz-Key, 1974), caused onchocerciasis of cattle and deer respectively (Adler 2004). Mermithids, able to decrease the population density of these vectors, and to affect species composition of the simuliid populations, could be considered as an essential epidemiology factor of these diseases.
